# Concentration and Application Timing Modulate Growth and Biochemical Composition of *Limnospira platensis* Exposed to Multipod ZnO Nanostructures

**DOI:** 10.3390/life16101604

**Published:** 2026-09-25

**Authors:** Tatiana Chiriac, Ludmila Rudi, Liliana Zosim, Tudor Braniște, Liliana Cepoi

**Affiliations:** 1 Institute of Microbiology and Biotechnology, Technical University of Moldova, MD 2028 Chisinau, Moldova; tatiana.chiriac@imb.utm.md (T.C.); ludmila.rudi@imb.utm.md (L.R.); liliana.zosim@imb.utm.md (L.Z.); 2 National Center for Materials Study and Testing, Technical University of Moldova, MD 2045 Chisinau, Moldova; tudor.braniste@cnstm.utm.md

**Keywords:** *Limnospira platensis*, ZnO multipod nanostructures, application timing, biochemical response, cyanobacteria

## Abstract

Zinc oxide nanostructures can induce highly variable biological responses depending on their physicochemical properties, concentration, and exposure conditions. This study investigated the effects of multipod ZnO nanostructures (ZnO-MPs) on *Limnospira platensis*, considering both concentration and application timing. ZnO-MPs (0.01–30 mg/L) were applied at 0, 72, or 120 h after inoculation, and the final biomass and the contents of proteins, carbohydrates, lipids, phycobiliproteins, phycocyanin, chlorophyll, and carotenoids were determined. Final biomass showed relatively limited and non-monotonic variation, whereas biochemical composition was substantially altered. Low concentrations (0.1–1 mg/L), particularly when applied at inoculation, stimulated total phycobiliprotein and phycocyanin accumulation. Higher concentrations strongly reduced protein and phycobiliprotein contents, while promoting carbohydrate and/or lipid accumulation under specific application conditions. Chlorophyll and carotenoids exhibited comparatively moderate and non-monotonic responses. Overall, the results demonstrate that the effects of ZnO-MPs on *Limnospira platensis* are strongly modulated by concentration and application timing, with biochemical responses being more pronounced than changes in final biomass. These findings emphasize the importance of considering both nanomaterial concentration and application timing when evaluating nanomaterial–cyanobacterium interactions.

## 1. Introduction

Nanomaterials remain a major focus of research because of their unique physicochemical properties and broad range of technological and biological applications [1,2]. Their properties and functionality are strongly influenced by characteristics such as size, dimensionality, and morphology [2]. These characteristics can also influence the behavior of nanomaterials and their interactions with biological systems [1]. Consequently, evaluating the biological effects of nanomaterials requires consideration not only of the exposure concentration but also of their physicochemical characteristics and the specific features of the exposed biological system.

Metal oxide nanoparticles are an important category of nanomaterials because of their distinct physicochemical and biological properties [3]. Among them, zinc oxide nanoparticles (ZnONPs) are of particular interest, and their biological activity is influenced by particle size, morphology, surface properties, crystal structure, aggregation state, and particle stability [4]. ZnONPs can form a wide variety of nanoscale shapes and architectures, ranging from spherical particles and one-dimensional structures to complex three-dimensional configurations [3]. Morphology is of particular interest because particle geometry can alter the surface area available for interaction, aggregation behavior, and contact with the cell surface, thereby influencing the intensity and nature of the biological response.

Previous studies indicate that the biological effects of ZnO nanostructures can vary with particle morphology, although the direction and magnitude of this effect depend on the biological system and particle dimensions. Peng et al. (2011) compared spherical ZnO nanoparticles (6.3–15.7 nm) with one-dimensional rod- and needle-like structures (242–862 nm) at 10–80 mg/L in marine diatoms and found the one-dimensional structures to be more toxic than spheres to *Phaeodactylum tricornutum* [5]. In contrast, Samei et al. (2019), using *Raphidocelis subcapitata*, reported greater cell mortality for spherical than rod-shaped ZnO at 0.7 mg/L (approximately 50% versus 30%, respectively), while increasing nanorod length from 100 to 500 nm markedly reduced toxicity [6]. Pereira et al. (2020) further showed morphology-dependent responses in *Pseudokirchneriella subcapitata*, with flower-like ZnO structures exhibiting greater toxicity than coprecipitated and nanoplate-like materials [7]. Together, these findings indicate that ZnO morphology can modulate algal responses but that its effect is context-dependent rather than universally associated with a particular particle shape. This context motivated the investigation of multipod ZnO nanostructures as a distinct branched hierarchical architecture in a cyanobacterial system. The present study therefore examines the biological response of *L. platensis* to this specific ZnO architecture, without assuming that the observed effects are attributable exclusively to morphology.

Microalgae and cyanobacteria are particularly relevant photosynthetic systems for studying interactions with nanomaterials. In addition to their ecological importance, they have considerable biotechnological value due to their high productivity and capacity to synthesize lipids [8], carbohydrates [9], proteins [10], and pigments [11]. Their biomass is used in the food, pharmaceutical, and cosmetic industries; in agriculture and aquaculture; and for biofuel production [12,13,14,15,16,17,18,19]. Cyanobacteria are also recognized as sources of diverse bioactive metabolites, including pigments and lipid-derived compounds with potential antimicrobial and other biotechnological applications [20,21]. Because these biomass components have distinct metabolic functions and may respond differently to external factors, nanomaterial effects are relevant not only to culture growth but also to changes in biochemical composition and cellular function.

The interaction of nanoparticles with microalgae and cyanobacteria can generate highly variable responses depending on nanoparticle properties, concentration, exposure duration, cultivation conditions, and organism-specific characteristics [22,23]. For ZnONPs, substantial differences among microalgal species have been reported, along with effects dependent on particle size, concentration, and contact duration [24]. Moreover, the induced responses are not limited to changes in culture growth. Exposure to nanoparticles can alter the accumulation of proteins, carbohydrates, lipids, and pigments, indicating selective effects on different components of cellular metabolism [25]. Therefore, changes in biomass do not always capture the full magnitude of the biological response, and assessing multiple biochemical endpoints simultaneously may provide a more comprehensive characterization of nanomaterial effects on photosynthetic organisms.

Another potentially important determinant is the physiological context in which nanomaterial exposure begins. In the lag, exponential, and stationary phases, cells differ in growth rate, biosynthetic activity, resource allocation, and their capacity to adapt to stress factors. Consequently, the biological outcome of nanomaterial exposure may vary depending on when exposure is initiated during culture development. However, compared with the effects of concentration, particle size, and exposure duration, the influence of the application timing has been investigated less systematically in microalgae and cyanobacteria. Considering this factor may help explain some of the variability in responses to nanomaterials reported in the literature and clarify how the biological outcome varies according to the timing of exposure during culture development.

In this context, *Limnospira platensis* (formerly *Arthrospira platensis*, commonly known as spirulina) represents a suitable biological system for investigating complex responses to ZnO nanostructures. This cyanobacterium is widely used in biotechnological research and is cultivated on a large scale to produce commercially valuable biomass [26,27]. Its biomass is characterized by a high protein content and contains carbohydrates, lipids, and valuable pigments, particularly phycobiliproteins [28,29]. This diverse biochemical composition allows the simultaneous evaluation of multiple biomass components with potentially different sensitivities to nanomaterial exposure. This feature makes it possible to distinguish effects on overall biomass accumulation from selective changes in biochemical composition and, consequently, to characterize the biological response to ZnO nanostructures more comprehensively.

Previous studies have mainly addressed the effects of ZnO nanomaterials on microalgae and cyanobacteria as a function of concentration, whereas considerably less attention has been given to the timing of nanomaterial application during culture development. The present study addresses this gap by considering application timing, together with concentration, as an experimental determinant of the final response to ZnO multipod nanostructures. By simultaneously assessing biomass and several major biochemical components, this study further examines whether growth and biomass composition respond similarly or differentially to ZnO-MP exposure.

In the present study, ZnO nanostructures with a multipod morphology (ZnO-MPs) were synthesized and characterized, and their effects on *L. platensis* were evaluated using an experimental design integrating two exposure-related factors: nanostructure concentration and application timing. ZnO-MPs were applied over a broad concentration range at 0 h (lag phase), 72 h (exponential phase), and 120 h (stationary phase), and the biological response was assessed in terms of biomass accumulation and the contents of proteins, carbohydrates, lipids, total phycobiliproteins, phycocyanin, chlorophyll, and carotenoids. This study aimed to determine how ZnO-MP concentration and application timing modulate the response of *L. platensis* and to assess differences in sensitivity between overall biomass accumulation and the responses of major biochemical components.

## 2. Materials and Methods

### 2.1. Synthesis of ZnO Multipod Nanostructures

Zinc oxide multipod nanostructures (ZnO-MPs) were synthesized using a low-temperature aqueous hydrothermal method. Zinc nitrate hexahydrate (Zn(NO_3_)_2_·6H_2_O) and potassium hydroxide (KOH) were used as precursor materials, while deionized water served as the reaction medium. Briefly, 9.6 g of KOH was dissolved in 40 mL of deionized water under continuous mechanical stirring (solution A). Separately, 6.0 g of Zn(NO_3_)_2_·6H_2_O was dissolved in 40 mL of deionized water (solution B). Solution B was then added dropwise to solution A under continuous stirring. The initial formation of a milky-white Zn(OH)_2_ precipitate was followed by its gradual dissolution upon continued stirring for approximately 20 min, resulting in a clear solution attributed to the formation of soluble tetrahydroxozincate ions, [Zn(OH)_4_]^2−^.

The reaction mixture was subsequently maintained at 85 °C for 3 h under gentle stirring. After completion of the reaction, the suspension was rapidly cooled, frozen at −20 °C for 24 h, and subsequently lyophilized to obtain the ZnO powder used for characterization and biological experiments.

### 2.2. Morphological and Structural Characterization of ZnO Multipod Nanostructures

The morphology of the synthesized ZnO nanostructures was examined by scanning electron microscopy (SEM) at the “Petru Poni” Institute of Macromolecular Chemistry (ICMPP), Iași, Romania. Images were acquired in secondary-electron (SE) mode using an Everhart–Thornley detector (ETD), at an accelerating voltage of 20 kV and a beam current of 0.40 nA. Micrographs were recorded at magnifications ranging from 10,000× to 50,000×, with working distances of 5.2–5.7 mm. The morphology and dimensions of the elongated structural units and multipod assemblies were evaluated from calibrated SEM micrographs.

The crystalline structure and phase composition of the synthesized ZnO-MPs were examined by X-ray diffraction (XRD). Diffraction patterns were recorded in configuration theta/2theta 20–80°, PB, 5 mm slitover using a SmartLab, Rigaku, Japan with Cu Kα, λ = 1.5406 Å radiation. The diffraction peaks were indexed by comparison with the standard diffraction pattern of hexagonal wurtzite ZnO (JCPDS card No. 36-1451).

### 2.3. Release of Ultrafilterable Zn from ZnO-MPs in Culture Medium

Stock suspensions of ZnO-MPs (1 mg/mL) were prepared in sterile deionized water. The suspensions were sonicated in an ultrasonic bath (SONOREX DIGITEC DT, BANDELIN electronic GmbH & Co. KG, Berlin, Germany; 35 kHz) for 25 min at 20 °C and vortex-mixed for 30 s immediately before use. Appropriate volumes were transferred into sterile mineral culture medium to obtain final ZnO-MP concentrations of 5, 10, 20, and 30 mg/L. The suspensions were incubated in the absence of microorganisms under the same physicochemical conditions used for *L. platensis* cultivation. At 1, 6, 24, 72, and 120 h, aliquots were centrifuged at 11,000× *g* for 40 min at 20 °C. The resulting supernatants were subsequently processed using 3 kDa centrifugal ultrafiltration units (Sartorius, Göttingen, Germany) to separate particulate and colloidal Zn-containing material from the ultrafilterable Zn fraction. The <3 kDa fraction was operationally defined as dissolved Zn and quantified by flame atomic absorption spectrometry (FAAS) at 213.8 nm using certified Zn standard solutions. Zn release was expressed as the concentration of ultrafilterable Zn (mg/L) and as the percentage of the total Zn initially present in the added ZnO-MPs. The initial Zn content was calculated from the Zn mass fraction in ZnO. Data are presented as mean ± SD (*n* = 5 independent samples).

### 2.4. Limnospira platensis Strain, Mineral Medium, and Cultivation Conditions

The cyanobacterial strain *Limnospira platensis* CNMN-CB-02, obtained from the National Collection of Non-Pathogenic Microorganisms of the Institute of Microbiology and Biotechnology, Technical University of Moldova, was used in this study. The strain was cultivated for 6 days in a mineral medium with the following composition: NaNO_3_, 2.5 g/L; NaHCO_3_, 8.0 g/L; NaCl, 1.0 g/L; K_2_SO_4_, 1.0 g/L; Na_2_HPO_4_, 0.2 g/L; MgSO_4_·7H_2_O, 0.2 g/L; H_3_BO_3_, 2.86 mg/L; MnCl_2_·4H_2_O, 1.81 mg/L; CuSO_4_·5H_2_O, 0.08 mg/L; MoO_3_, 0.015 mg/L; and FeEDTA, 1 mL/L. The salts used for medium preparation were purchased from Sigma-Aldrich (Merck KGaA, Darmstadt, Germany). Cyanobacterial cultures were grown in 250 mL Erlenmeyer flasks with a working volume of 100 mL. Cultivation was carried out at 28 ± 1 °C, pH 8–10, and a light intensity of 37–55 µmol photons m^−2^ s^−1^ with periodic agitation [30].

### 2.5. Preparation of ZnO-MP Suspensions and Experimental Design

A stock suspension of ZnO-MPs (1 mg/mL) was freshly prepared by dispersing the nanostructure powder in deionized water. The suspension was subjected to ultrasonic treatment for 5 min at 20 °C in an ultrasonic bath (SONOREX DIGITEC DT, BANDELIN electronic GmbH & Co. KG, Berlin, Germany; 35 kHz). The freshly prepared suspension was immediately added to the *Limnospira platensis* cultures in appropriate volumes to obtain final ZnO-MP concentrations of 0.01, 0.1, 0.5, 1.0, 5.0, 10.0, 20.0, and 30.0 mg/L.

The concentration range (0.01–30 mg/L) was selected to cover several orders of magnitude and thereby capture both low-dose responses and potential effects at higher ZnO-MP levels. The range was intended primarily for concentration–response assessment under controlled cultivation conditions rather than to reproduce specific environmental exposure scenarios.

To evaluate the influence of ZnO-MP application timing during culture development, the nanostructures were added at three defined time points: immediately after inoculation (0 h), 72 h after inoculation, or 120 h after inoculation. Based on experimentally determined growth kinetics of untreated *L. platensis* cultures under the same cultivation conditions (*n* = 5 biological replicates; Figure S1 and Table S1), these time points corresponded to the lag, exponential, and stationary phases, respectively. Cultures without ZnO-MPs served as controls and were maintained under identical experimental conditions. All cultures were harvested at the common endpoint of 144 h (6 days), when biomass was collected for quantification and subsequent biochemical analyses.

### 2.6. Biomass Determination and Sample Preparation for Biochemical Analyses

Biomass concentration was estimated spectrophotometrically according to the approach described by Rudi et al. (2025) [30]. At the end of the 6-day cultivation period, *Limnospira platensis* cultures were homogenized, and absorbance was recorded at 680 nm using a T80 UV/VIS spectrophotometer (PG Instruments, Alma Park, Leicestershire, UK). Culture suspensions were serially diluted with purified water, when necessary, to maintain absorbance values within the linear range of 0.1–0.4. To account for the potential optical contribution of ZnO-MPs, a concentration-matched cell-free blank was used for each ZnO-MP treatment. Each blank consisted of culture medium containing the same ZnO-MP concentration as the corresponding experimental sample and was subjected to the same dilution prior to spectrophotometric measurement. For untreated control cultures, ZnO-MP-free culture medium was used as the blank. Biomass concentration, expressed as dry weight (g/L), was calculated using a calibration curve relating absorbance at 680 nm to biomass dry weight.

For biochemical analyses, biomass from the control and experimental cultures was separated from the culture medium by vacuum filtration through Whatman Grade 1 qualitative filter paper. The retained biomass was gently mixed with a 2% ammonium acetate solution for demineralization and subjected to a second filtration step. The biomass was then washed with double-distilled water to remove residual salts and carefully recovered from the filter. The recovered biomass was resuspended in distilled water and adjusted to a concentration of 10 mg/mL. To facilitate cell disruption and the release of intracellular components, the samples were subjected to six consecutive freeze–thaw cycles, each consisting of freezing at −20 °C followed by thawing at room temperature. The prepared samples were stored at −20 °C until biochemical analysis.

### 2.7. Biochemical Analysis

The reagents used for the biochemical analyses were obtained from Sigma-Aldrich (Merck KGaA), Darmstadt, Germany.

#### 2.7.1. Determination of Protein Content

Protein content was determined using the Lowry method, with adaptations for the biological material analyzed [31]. An alkaline extract was prepared by mixing 0.1 mL of the standardized biomass suspension (10 mg/mL) with 0.9 mL of 0.1 N NaOH and incubating the mixture at room temperature for 30 min. An aliquot (0.2 mL) of the resulting mixture was combined with 0.8 mL of distilled water and 1.5 mL of alkaline copper reagent. The reagent was prepared by mixing 2% Na_2_CO_3_ in 0.1 N NaOH with 0.5% CuSO_4_ in 1% sodium potassium tartrate at a 49:1 (*v*/*v*) ratio. After homogenization and incubation at room temperature for 10 min, 0.5 mL of 0.1 N Folin–Ciocalteu reagent was added. The samples were homogenized and incubated in the dark at room temperature for 40 min, after which the absorbance was measured at 750 nm. Protein content was calculated using a calibration curve constructed with bovine serum albumin (BSA) and expressed as a percentage of biomass dry weight.

#### 2.7.2. Determination of Carbohydrate Content

Carbohydrate content was determined using the anthrone method [32]. The standardized biomass suspension (10 mg/mL) was diluted tenfold, and 0.2 mL of the diluted sample was mixed with 2.0 mL of anthrone reagent prepared by dissolving 50 mg of anthrone in 100 mL of 66% H_2_SO_4_. The samples were then homogenized and incubated at room temperature for 10 min, followed by incubation in a water bath (Gesellschaft für Labortechnik mbH, Burgwedel, Germany) at 100 °C for 10 min. After cooling under running cold water, the samples were kept in the dark at room temperature for 30 min, after which the absorbance was measured at 620 nm. Carbohydrate content was calculated using a calibration curve constructed using D-glucose as a standard and expressed as a percentage of biomass dry weight.

#### 2.7.3. Determination of Lipid Content

Lipid content was determined using the sulfo-phospho-vanillin method [33]. Lipids were extracted from the biomass as follows. A 1.0 mL aliquot of the standardized biomass suspension (10 mg/mL) was centrifuged, and the resulting pellet was treated with 1.0 mL of a chloroform–ethanol mixture (2:1, *v*/*v*). The samples were continuously agitated (Unimax 1010, Heidolph Scientific Products GmbH, Schwabach, Germany) at room temperature for 120 min. After separation of the lipid extract from the biomass, the solvent was completely evaporated. The resulting lipid residue was treated with 1.0 mL of concentrated H_2_SO_4_, incubated in a water bath at 90 °C for 20 min, and subsequently cooled to room temperature. An aliquot (0.1 mL) of the resulting mixture was mixed with 3.0 mL of phospho-vanillin reagent containing 1.2 mg/mL vanillin in 68% H_3_PO_4_. Color development was allowed to proceed for 30 min at room temperature in the dark, after which the absorbance was measured at 520 nm. Lipid content was calculated from a calibration curve constructed using oleic acid as a standard and expressed as a percentage of biomass dry weight.

#### 2.7.4. Chlorophyll and Carotenoid Content Determination

Chlorophyll and total carotenoid contents were determined spectrophotometrically using ethanolic pigment extracts. A 1.0 mL aliquot of the standardized biomass suspension (10 mg/mL) was centrifuged (MIKRO 22R, Andreas Hettich GmbH & Co. KG, Tuttlingen, Germany), and the supernatant was discarded. The resulting pellet was resuspended in 1.0 mL of 96% ethanol. Pigments were extracted under continuous agitation at room temperature for 12 h. The samples were then centrifuged, and the supernatant was collected for analysis. For spectrophotometric measurements, 0.1 mL of the ethanolic extract was diluted with 3.0 mL of 96% ethanol. Absorbance was measured at 450, 649, and 665 nm. Chlorophyll a and total carotenoid concentrations were calculated using the equations proposed by Lichtenthaler and Wellburn (1983) [34]:

(1) Chlorophyll a (mg/mL) = (13.36 × A_665_ − 5.19 × A_649_)/1000

(2) Total carotenoids (mg/mL) = (1000 × A_450_ − 2.05 × chlorophyll *a*)/221

Pigment contents were expressed as percentages of biomass dry weight.

#### 2.7.5. Phycobiliprotein Content Determination

To prepare the aqueous phycobiliprotein extract, 1.0 mL of the standardized biomass suspension (10 mg/mL) previously subjected to six freeze–thaw cycles was centrifuged and the resulting supernatant was used for spectrophotometric analysis. Absorbance was measured at 620 and 650 nm. Phycocyanin (PC) and allophycocyanin (APC) concentrations were calculated according to the equations described by Siegelman et al. (1978) [35]:

(3) Phycocyanin (PC) (mg/mL) = [A_620_ − (0.7 × A_650_)]/7.3

(4) Allophycocyanin (APC) (mg/mL) = [A_650_ − (0.208 × A_620_)]/5.09

Total phycobiliprotein content was calculated as the sum of phycocyanin and allophycocyanin concentrations (PC + APC). Phycocyanin and total phycobiliprotein contents were expressed as percentages of biomass dry weight.

#### 2.7.6. Determination of Malondialdehyde Content

Malondialdehyde (MDA) content was determined using the thiobarbituric acid (TBA) reactive substances assay, as previously described [30]. Briefly, 10 mg of biomass was mixed with 3.0 mL of 0.76% TBA prepared in 20% trichloroacetic acid. The reaction mixture was incubated at 95 °C for 20 min and subsequently cooled to room temperature. Absorbance was measured at 532 and 600 nm, with the latter used for correction of non-specific absorbance. MDA content was calculated from the corrected absorbance using the molar extinction coefficient of the MDA–TBA complex and expressed as nM g^−1^ biomass.

#### 2.7.7. Determination of Antioxidant Activity by the ABTS Assay

Antioxidant activity was determined using the ABTS radical cation (ABTS^•+^) scavenging assay. The ABTS^•+^ solution was generated by mixing 7 mM ABTS with 2.45 mM potassium persulfate at a 1:1 (*v*/*v*) ratio and allowing the mixture to react in the dark at room temperature for 12–16 h. Before analysis, the solution was diluted to an absorbance of 0.700 ± 0.020 at 734 nm. For the assay, 0.3 mL of aqueous biomass extract was mixed with 2.7 mL of the ABTS^•+^ working solution. After 6 min of incubation, absorbance was recorded at 734 nm. Antioxidant activity was calculated as the percentage inhibition of ABTS^•+^ relative to the reagent control [36].

### 2.8. Statistical Analysis

Experiments were conducted with five independent biological replicates (*n* = 5) per experimental condition, and results were reported as mean ± standard deviation (SD). Technical measurements were not treated as independent biological replicates. Differences among ZnO-MP concentrations within each application timing (0, 72, or 120 h after inoculation) were assessed using one-way analysis of variance (one-way ANOVA), followed by Tukey’s post hoc test for multiple comparisons. To assess the effects of ZnO-MP concentration, the application timing, and the interaction between these two factors on the parameters analyzed, a two-way analysis of variance (two-way ANOVA) was performed. Each biological endpoint was analyzed separately, and correction for multiple pairwise comparisons within each analysis was performed using Tukey’s HSD test. Differences were considered statistically significant at *p* < 0.05. Statistical analyses were performed using IBM SPSS Statistics, version 19.0 (IBM Corp., Armonk, NY, USA).

## 3. Results

### 3.1. Morphological Characteristics of the Synthesized ZnO Multipod Nanostructures

SEM analysis revealed a characteristic hierarchical morphology of the synthesized ZnO-MPs (Figure 1a–c). At higher magnifications, the material consisted of numerous elongated rod-/lance-like units arranged predominantly in radial configurations, forming well-defined three-dimensional flower-/rosette-like multipod structures. The elongated units were estimated to be approximately 0.4–0.9 μm in length and 0.10–0.20 μm in width, with most structures falling within the ranges of 0.5–0.8 μm and 0.12–0.18 μm, respectively. Individual multipod assemblies were typically 1–3 μm in overall size and were further associated into larger irregular aggregates. The recurrence of this architecture across different fields of view indicated that the multipod morphology was a characteristic feature of the synthesized ZnO material rather than a localized morphological pattern.

XRD analysis further confirmed the crystalline structure of the ZnO-MPs (Figure 1d). Diffraction peaks were observed at 2θ = 31.7°, 34.4°, 36.2°, 47.5°, 56.6°, 62.8°, 66.3°, 67.9°, and 69.1°, corresponding to the (100), (002), (101), (102), (110), (103), (200), (112), and (201) crystallographic planes, respectively. All reflections were indexed to the hexagonal wurtzite structure of ZnO (space group P6_3_mc; JCPDS card No. 36-1451). No additional diffraction peaks attributable to secondary crystalline phases were detected, supporting the phase purity of the synthesized ZnO-MPs. The sharp and well-defined reflections further indicate the crystalline nature of the material.

### 3.2. Release of Ultrafilterable Zn from ZnO-MPs in Culture Medium

The release of ultrafilterable Zn from ZnO-MPs was evaluated in cell-free culture medium under the same physicochemical conditions used for L. platensis cultivation (Table S2). The ultrafilterable Zn concentration remained essentially unchanged throughout the investigated period (1–120 h), indicating no measurable progressive release over time. Mean ultrafilterable Zn concentrations were 0.366 ± 0.005, 0.468 ± 0.016, 0.514 ± 0.005, and 0.556 ± 0.005 mg/L for 5, 10, 20, and 30 mg/L ZnO-MPs, respectively. When expressed relative to the total elemental Zn initially present in the ZnO-MPs, the ultrafilterable fraction decreased progressively with increasing ZnO-MP concentration, from 9.11 ± 0.14% at 5 mg/L to 5.83 ± 0.20%, 3.20 ± 0.03%, and 2.31 ± 0.02% at 10, 20, and 30 mg/L, respectively. Thus, despite a six-fold increase in ZnO-MP concentration, the absolute concentration of ultrafilterable Zn increased only modestly, indicating limited Zn release under the alkaline cultivation conditions.

### 3.3. Effects of ZnO-MP Concentration and Application Timing on Biomass and Protein Content

Application of ZnO-MPs produced statistically significant changes in final biomass; however, their magnitude was relatively small compared with the changes observed in biochemical composition. Overall, biomass remained within the range of 0.841–0.975 g/L, with no progressive inhibition as ZnO-MP concentration increased (Figure 2a). Following application at 0 h, biomass decreased moderately at 0.01–1 mg/L, returned to the control level at 5 mg/L, and reached a maximum at 10 mg/L (0.9746 ± 0.0100 g/L), approximately 7% above the control (0.9108 ± 0.0152 g/L). At 30 mg/L, biomass decreased to 0.8586 ± 0.0231 g/L. Following application at 72 h after inoculation, the lowest value was recorded at 5 mg/L (0.8412 ± 0.0087 g/L), whereas at 10–20 mg/L, biomass approached the control level. Following application at 120 h after inoculation, the response was also non-monotonic, with a maximum at 0.5 mg/L (0.9390 ± 0.0195 g/L) and a minimum at 30 mg/L (0.8406 ± 0.0136 g/L). These results indicate statistically significant but quantitatively limited changes in final biomass and the absence of a monotonic concentration-dependent inhibition pattern.

Protein content showed a much more pronounced response and was strongly dependent on application timing (Figure 2b). Following application at 0 h, low and intermediate concentrations produced moderate variations, with a significant increase at 1 mg/L (70.92 ± 1.14% of biomass), followed by progressive reductions at 10–30 mg/L, reaching 46.20 ± 0.84% at the highest concentration. After application at 72 h, protein content increased at 0.5 and 5 mg/L, reaching 73.68 ± 0.76% and 72.84 ± 1.03%, respectively, before decreasing at higher concentrations; at 30 mg/L, the value was 53.76 ± 0.81%. Application of ZnO-MPs at 120 h resulted in the most pronounced decrease: the effect became evident at 5 mg/L (41.10 ± 0.88%), while at 10–30 mg/L, protein content remained at approximately 28.7–32.7%, corresponding to reductions of approximately 52–58% relative to the control.

Two-way ANOVA confirmed significant effects of concentration, application timing, and their interaction on both biomass and protein content (*p* < 0.001; Table 1). The interaction was particularly evident for protein content, with high concentrations producing a substantially stronger reduction when applied at 120 h after inoculation.

### 3.4. Effects of ZnO-MP Concentration and Application Timing on Carbohydrate and Lipid Contents

Carbohydrate content responded differently depending on ZnO-MP application timing (Figure 3a). After application at 0 h, carbohydrate content increased from 9.54 ± 0.15% in the control to a maximum of 11.61 ± 0.15% at 0.1 mg/L and then decreased at higher concentrations to 7.47 ± 0.19% at 30 mg/L. An almost opposite profile was observed following application at 72 h after inoculation. After a decrease at 0.01 mg/L, carbohydrate content increased in most treatments, reaching 13.46 ± 0.10% at 10 mg/L and 13.33 ± 0.09% at 30 mg/L, approximately 40–41% above the control. Following application at 120 h after inoculation, the response was less consistent; values generally remained close to or below the control level, with the minimum recorded at 30 mg/L (7.85 ± 0.15%). Differences among application timings became particularly evident at higher concentrations, where carbohydrate accumulation was predominantly enhanced following application at 72 h after inoculation.

Lipid content showed moderate changes at concentrations up to 5 mg/L and a sharp increase at higher concentrations, depending on application timing (Figure 3b). Following application at the inoculation time, values ranged from 3.88 to 4.98% of biomass, with significant differences among some concentrations but no monotonic trend. Following application at 72 h after inoculation, 0.5 and 5 mg/L moderately reduced lipid content, whereas 10–30 mg/L markedly increased it, reaching 8.65 ± 0.10% at 10 mg/L and 11.86 ± 0.19% and 11.78 ± 0.29% at 20 and 30 mg/L, respectively. Pronounced increases were also observed following application at 120 h after inoculation, where 10–30 mg/L resulted in values of 9.44–10.21%, more than twice the control level (4.47 ± 0.19%).

For both parameters, ZnO-MP concentration, application timing, and their interaction had significant effects (*p* < 0.001; Table 1). The interaction was reflected in divergent responses at higher concentrations: carbohydrate accumulation was predominantly enhanced after application at 72 h, whereas lipid accumulation was strongly increased after application at both 72 and 120 h.

### 3.5. Effects of ZnO-MP Concentration and Application Timing on Phycobiliprotein and Phycocyanin Contents

Total phycobiliproteins exhibited a non-monotonic concentration–response pattern, with increased contents at low concentrations and pronounced reductions at high concentrations (Figure 4a). Following application at inoculation time, variations were relatively moderate up to 5 mg/L, with slightly increased values at 0.1 and 5 mg/L, after which total phycobiliprotein content decreased to 14.19 ± 0.53% at 10 mg/L, 11.62 ± 0.33% at 20 mg/L, and 5.17 ± 0.16% at 30 mg/L. Following application at 72 h after inoculation, concentrations of 0.1–1 mg/L increased the content to 20.35–20.88%, compared with 17.28 ± 0.17% in the control, whereas 5–30 mg/L resulted in a progressive decrease, reaching 1.44 ± 0.12% at 30 mg/L. The response was even more pronounced following application at 120 h after inoculation: 0.1–1 mg/L increased phycobiliprotein content to 20.10–20.78%, whereas values decreased to 4.20 ± 0.27% at 5 mg/L and to below 1% at 10–30 mg/L, reaching 0.34 ± 0.09% at the highest concentration.

Phycocyanin followed a similar pattern but showed even greater sensitivity to high concentrations. Following application at 0 h, 0.01 mg/L increased phycocyanin content to 10.40 ± 0.10%, compared with 9.28 ± 0.07% in the control, whereas at 20 and 30 mg/L, the content decreased to 6.37 ± 0.24% and 3.53 ± 0.13%, respectively (Figure 4b). Following application at 72 h after inoculation, concentrations of 0.1–1 mg/L markedly increased phycocyanin content, with values ranging from 12.74 to 12.94%, followed by a sharp decrease to 6.81 ± 0.15% at 5 mg/L, approximately 3.2–3.3% at 10–20 mg/L, and 0.167 ± 0.012% at 30 mg/L. Following application at 120 h, the maximum value was recorded at 0.5 mg/L (13.66 ± 0.13%), followed by 13.33 ± 0.08% at 1 mg/L. Above this concentration range, phycocyanin content decreased to 2.23 ± 0.18% at 5 mg/L and 0.17 ± 0.08% at 30 mg/L.

Two-way ANOVA revealed highly significant effects of ZnO-MP concentration, application timing, and their interaction on both total phycobiliprotein and phycocyanin contents (*p* < 0.001; Table 1).

### 3.6. Effects of ZnO-MP Concentration and Application Timing on Chlorophyll and Carotenoid Contents

Chlorophyll content exhibited a non-monotonic response that depended on the application timing. Following application at inoculation time, values fluctuated around the control level (1.331 ± 0.015%): 0.01 mg/L resulted in a significant increase to 1.466 ± 0.023%, whereas at 0.5 mg/L, the content decreased to 1.289 ± 0.021%; at 30 mg/L, it increased again to 1.460 ± 0.022% (Figure 5a). Following application at 72 h, concentrations of 0.01–1 mg/L reduced chlorophyll content, with the greatest decrease observed at 1 mg/L (1.077 ± 0.018%, approximately 19% below the control). At 30 mg/L, this trend was reversed, with the value increasing to 1.491 ± 0.069%. Following application at 120 h, increases were observed at certain concentrations: 0.1 mg/L increased chlorophyll content to 1.530 ± 0.015%, while 20 and 30 mg/L increased it to 1.510 ± 0.014% and 1.536 ± 0.016%, respectively.

Carotenoid content was less affected following application at inoculation time, with values remaining within a narrow range (0.213–0.232% of biomass). Following application at 120 h after inoculation, variations were also moderate, although a significant decrease to 0.206 ± 0.008% was observed at 5 mg/L compared with 0.229 ± 0.008% in the control (Figure 5b). The most pronounced response occurred after application at 72 h after inoculation, when most values were higher than the control; at 30 mg/L, carotenoid content reached 0.331 ± 0.050%, corresponding to an increase of approximately 44.5%.

ZnO-MP concentration, application timing in relation to the growth phase, and their interaction significantly affected both chlorophyll and carotenoid contents (*p* < 0.001; Table 1). Chlorophyll exhibited a strongly non-monotonic response, whereas increases in carotenoid content were observed predominantly following application 72 h after inoculation.

### 3.7. Effects of ZnO-MP Concentration and Application Timing on Lipid Peroxidation and Antioxidant Activity

Malondialdehyde (MDA) content showed a concentration- and application timing-dependent response to ZnO-MP exposure (Figure 6a). Following application at the onset of the lag phase, MDA remained close to or slightly below the control level (40.18 ± 1.90 nM/g) at concentrations up to 0.5 mg/L. A significant increase became evident from 1 mg/L, with MDA reaching 51.95 ± 2.03 nM/g at 10 mg/L and approximately 62–64 nM/g at 20–30 mg/L. Following application during the exponential phase, MDA initially decreased below the control level at low ZnO-MP concentrations and subsequently increased progressively, reaching 66.54 ± 1.14 nM/g at 30 mg/L. The response was particularly pronounced following application during the stationary phase, where MDA increased progressively with concentration, reaching 67.87 ± 0.62 and 69.73 ± 1.90 nM/g at 20 and 30 mg/L, respectively.

Antioxidant activity of the aqueous biomass extracts, determined by the ABTS assay, showed an approximately opposite concentration-dependent pattern (Figure 6b). Following application during the lag phase, antioxidant activity increased slightly at 0.01–0.1 mg/L relative to the control (45.46 ± 0.64) but subsequently decreased with increasing ZnO-MP concentration, reaching 21.09 ± 0.58 at 30 mg/L. A similar pattern was observed following application during the exponential phase, with slight stimulation at the lowest concentrations, followed by a progressive decline at higher concentrations. The strongest reduction occurred following application during the stationary phase: antioxidant activity decreased markedly from 0.1 mg/L onward and reached 13.07 ± 0.50 at 30 mg/L.

Two-way ANOVA confirmed significant effects of ZnO-MP concentration, application timing, and their interaction on both MDA content and ABTS antioxidant activity (*p* < 0.001; Table 1). Overall, increasing ZnO-MP concentrations were associated with enhanced lipid peroxidation and reduced antioxidant activity, although the magnitude and onset of these responses depended strongly on application timing.

## 4. Discussion

### 4.1. Zn Release from ZnO-MPs Under Cultivation Conditions

The behavior of ZnO-MPs in the culture medium is relevant to the interpretation of their biological effects because ZnO can partially dissolve and release bioavailable Zn species. In the present study, however, Zn release was limited and showed no measurable progressive increase between 1 and 120 h. The ultrafilterable Zn fraction decreased from 9.11% of the initial Zn at 5 mg/L ZnO-MPs to 2.31% at 30 mg/L, while the corresponding absolute concentration increased only moderately, from approximately 0.37 to 0.56 mg/L.

ZnO dissolution is strongly influenced by the surrounding medium. In microalgal culture media, Yung et al. (2017) [37] showed that ZnO dissolution was affected by both medium composition and pH, with lower dissolved Zn concentrations observed at pH 8 than at pH 7. A greater fractional dissolution at lower initial ZnO concentrations has also been reported, while carbonate alkalinity has been identified as an important factor controlling ZnO dissolution [38]. These observations are consistent with the limited Zn release measured in the alkaline, bicarbonate-containing medium used for L. platensis cultivation in the present study.

Thus, extensive dissolution of ZnO-MPs is unlikely to account alone for the observed biological responses. Nevertheless, the <3 kDa fraction measured here represents operationally defined ultrafilterable Zn rather than specifically free Zn^2+^. Therefore, a contribution of dissolved Zn species to the biological effects cannot be excluded.

### 4.2. Overall Biological Response: Biochemical Remodeling Without Major Growth Inhibition

The results demonstrate that the response of *Limnospira platensis* to ZnO-MPs cannot be described by a simple dose–toxicity relationship. Final biomass was significantly influenced by ZnO-MP concentration, application timing, and interaction, although the magnitude of the observed changes remained relatively small. Biomass values ranged from approximately 0.841 to 0.975 g/L and did not show progressive inhibition with increasing concentration. Depending on application timing and concentration, both statistically significant increases and decreases were observed. For example, following application in the lag phase, biomass peaked at 10 mg/L, approximately 7% above the control.

The lowest biomass values, in contrast, were observed at 5 mg/L following application during the exponential phase and at 30 mg/L following application in the stationary phase. Despite this relatively limited quantitative variation in biomass, biochemical composition was altered to a much greater extent. This difference between the magnitude of the growth response and that of the biochemical response indicates that final biomass is a less sensitive endpoint for characterizing the effects of ZnO-MPs in the system investigated and, when considered alone, does not reflect the full extent of biochemical remodeling.

The non-monotonic responses observed for several endpoints may reflect a transition from adaptive or compensatory responses at lower exposure levels to metabolic impairment at higher exposure levels. Such biphasic patterns may be consistent with a hormesis-like response; however, the present data do not allow hormesis to be established mechanistically.

This variability is consistent with the available data on ZnONPs, for which biological effects differ depending on concentration, particle properties, exposure conditions, and organism-specific characteristics [22,25,39,40]. In cyanobacteria, spherical ZnONPs produced concentration-dependent responses in both *Arthrospira platensis* and *Nostoc linckia* [30,41]. At the same time, studies on microalgae have reported the maintenance or even stimulation of growth within certain concentration ranges in *Dunaliella salina*, *Scenedesmus rubescens*, *Lobosphaera* sp., and *Chlorella* sp., whereas higher concentrations resulted in inhibitory effects on growth and/or biochemical composition [42,43,44,45,46]. Under other experimental conditions, *Arthrospira platensis* showed substantial reductions in biomass and protein content following exposure to ZnONPs [47,48]. Taking together, these observations support the view that the effects of ZnONPs on photosynthetic organisms are conditioned by the biological system and exposure characteristics and that evaluating growth alone may underestimate early or selective metabolic responses.

The comparative discussion includes examples from both cyanobacteria and microalgae. *Limnospira platensis* is a cyanobacterium, and comparisons with other cyanobacteria are therefore biologically the most relevant. However, the literature explicitly addressing the influence of ZnO morphology and the physiological context of exposure on nanoparticle responses is more extensive for microalgae. These studies provide a relevant basis for comparison because microalgae and cyanobacteria are photosynthetic microorganisms cultivated in aquatic systems and are exposed to common processes involving particle–cell surface interactions, aggregation in the culture medium, and disruption of photosynthetic and metabolic processes. Nevertheless, taxonomic and structural differences between these two groups mean that such comparisons should be interpreted as contextual support rather than direct equivalents of the biological responses observed in cyanobacteria.

### 4.3. Morphology-Dependent Effects of ZnO Nanostructures: Comparison with Previous Studies

In addition to concentration, the size and morphology of ZnONPs can influence the magnitude of the biological response. In *Dunaliella tertiolecta*, particle size affected the concentration at which growth effects became evident, indicating that primary particle size and particle behavior in the culture medium can modify the effective exposure of cells [49]. Morphology adds another layer of complexity, as particle geometry can alter the available contact surface, aggregation behavior, and interactions with the cell surface.

The importance of morphology has been demonstrated in several microalgal systems. Peng et al. (2011) compared spherical ZnO particles, nanorods, and nanoneedles in three marine diatoms and found that growth inhibition varied according to both species and nanostructure geometry [5]. Samei et al. (2019), using *Raphidocelis subcapitata*, showed that spherical nanoparticles and short nanorods exerted more pronounced effects than long nanorods, while aggregation of spherical particles reduced the differences associated with primary particle size [6]. Similarly, Pereira et al. (2020) reported different effects of aggregated particles, nanoplates, and flower-like structures in *Pseudokirchneriella subcapitata*, with the flower-like structures producing the most pronounced effect [7]. In *Chlamydomonas monadina*, ZnO nanostructures with a lamellar morphology stimulated growth within an intermediate concentration range but produced adverse effects outside this range [50]. Although these studies are not directly comparable with the present experiment, they consistently demonstrate that the shape and structural organization of ZnO can influence its interactions with photosynthetic cells.

A particularly relevant reference is our previous study conducted on the same species, *Arthrospira platensis*, using commercial spherical ZnO NPs and a comparable concentration range [30]. In that study, exposure from the first day of cultivation reduced biomass by 37.7–55.8% at 10–20 mg/L and caused substantial decreases in protein and phycobiliprotein contents. In the present experiment, ZnO-MPs produced statistically significant changes in biomass when applied in the lag phase. However, these changes were considerably smaller in magnitude and did not follow a monotonic concentration-dependent inhibition pattern. At 10 mg/L, biomass was approximately 7% higher than the control, while at 30 mg/L, the reduction remained moderate. The difference between these response profiles suggests that the morphological characteristics of the nanostructures may contribute to the distinct response of *L. platensis*. The branched architecture of ZnO-MPs may modify their interaction with the filament surface through differences in particle geometry, aggregation behavior, and the extent and mode of particle-cell contact. Such physical interactions could contribute to the biological response observed here. However, specific surface area, aggregation in the presence of cells, and particle–cell interactions were not directly assessed. Moreover, because spherical particles and multipod nanostructures were not compared side by side under identical experimental conditions, morphology should be regarded as a plausible contributing factor rather than a demonstrated causal mechanism.

### 4.4. Application Timing Shapes the Response to ZnO-MPs

A key result of the present study is that the biological outcome measured at the common harvest point depended strongly on the timing of ZnO-MP application during culture development. The three application times corresponded experimentally to the lag, exponential, and stationary phases (Figure S1 and Table S1). However, because all cultures were harvested at the same endpoint, later application also resulted in progressively shorter exposure durations. Therefore, the differences among the three application timings should be interpreted as the combined consequence of the physiological context at exposure initiation and the subsequent duration of exposure, rather than as evidence of intrinsic phase-specific sensitivity.

The importance of the physiological state of the culture at the onset of exposure has also been demonstrated in other photosynthetic systems. In *Chaetoceros neogracile*, exposure to polystyrene nanoparticles produced more pronounced effects during the exponential phase than during the stationary phase, with these differences associated with both the culture’s physiological vulnerability and changes in particle behavior in the culture medium [51]. In *Chlamydomonas reinhardtii*, AgNPs exhibited substantially greater toxicity during the exponential phase than during later stages of the growth cycle [52]. In *Porphyridium cruentum*, application of AuNPs and AgNPs during the lag or exponential phase differentially affected growth dynamics and lipid accumulation [53]. Similarly, in *Haematococcus lacustris*, the same concentrations of ZnONPs produced different effects on lipid accumulation during the palmella and aplanospore stages [54]. Together, these examples support the general concept that the physiological state of the culture is an important determinant of the response to nanomaterials.

Importantly, in the experimental design of the present study, the timing of application simultaneously altered two variables: the culture’s physiological state and the total duration of contact with ZnO-MPs before harvesting. Therefore, the differences observed following application on days 1, 3, and 5 cannot be attributed exclusively to the physiological phase. Rather, they reflect the combined effects of the culture’s physiological state at the onset of exposure and the duration of exposure. This distinction is important for the biological interpretation of the results and highlights the need for future experiments in which exposure duration is controlled independently of the physiological stage at which exposure begins.

### 4.5. Differential Sensitivity of Proteins and Photosynthetic Pigments

Among the biomass components analyzed, proteins and phycobiliproteins showed the most pronounced sensitivity to ZnO-MPs. Protein content exhibited moderate variations and even some stimulation at low and intermediate concentrations, followed by reductions at higher concentrations. Following application in the lag and exponential phases, decreases became particularly evident at 10–30 mg/L. Application in the stationary phase produced a substantially stronger response: protein content decreased already at 5 mg/L, while at 10–30 mg/L, the reductions reached approximately 52–58% relative to the control. This contrast, together with the relatively limited quantitative changes in final biomass, suggests that protein content is considerably more responsive to ZnO-MPs than overall biomass accumulation.

Phycobiliproteins, particularly phycocyanin, exhibited an even more distinct response. Following application during the lag phase, concentrations of 0.01–5 mg/L produced only minor changes, whereas 10–30 mg/L progressively decreased the content. Application in the exponential and stationary phases, however, revealed a stimulatory range at 0.1–1 mg/L, with increases of approximately 17–21% in phycobiliprotein content and stimulation of phycocyanin accumulation, followed by a sharp decline at higher concentrations. At 30 mg/L, phycocyanin content was reduced by more than 95% relative to the control. Two-way ANOVA of phycocyanin content revealed significant effects of ZnO-MP concentration, application timing, and their interaction, indicating that the response cannot be attributed to either factor alone.

Previous studies also support the sensitivity of phycocyanin to ZnONPs. Spherical ZnONPs significantly reduced phycocyanin content in *Arthrospira platensis* and, at high concentrations, resulted in the complete loss of the pigment [30]. In *Nostoc linckia*, 10–30 mg/L spherical ZnONPs reduced phycobiliprotein content by up to approximately 40% [41]. Djearamane et al. (2018) initially observed a slight stimulation of phycocyanin after 6–12 h of exposure, followed by a significant reduction at all tested concentrations after 96 h [48]. This temporal pattern is consistent with the concept that phycocyanin can respond rapidly and dynamically to ZnONP exposure, with the direction of the response depending on exposure intensity and duration.

Compared with proteins and phycobiliproteins, chlorophyll and carotenoids exhibited more moderate, although significant and strongly application timing-dependent, changes. Chlorophyll showed a non-monotonic response: following application in the lag phase, both increases and decreases relative to the control were observed; in the exponential phase, the maximum reduction was approximately 19% at 1 mg/L, whereas at 30 mg/L the content increased again. Following application during the stationary phase, stimulation was evident at certain concentrations, including 20–30 mg/L, where the values were approximately 13.5–15.4% higher than the control. Carotenoid content was relatively unaffected after application in the lag and stationary phases, although significant differences were observed at individual concentrations; application in the exponential phase, by contrast, produced more pronounced stimulation of carotenoid accumulation, reaching an increase of approximately 44.5% at 30 mg/L. A comparable response, characterized by maintenance of pigment levels at low concentrations and their reduction at higher concentrations, was reported in *Lobosphaera* sp. [46]. In the present system, the absence of a generalized decline in chlorophyll and carotenoids, even when phycobiliproteins and proteins were markedly reduced, indicates differential sensitivity among the components of the photosynthetic apparatus.

### 4.6. Carbon Allocation and Metabolic Reprogramming Under ZnO-MP Exposure

Changes in carbohydrate and lipid contents further support the pattern of selective metabolic reorganization. After application in the lag phase, carbohydrate accumulation increased at low concentrations, peaking at 0.1 mg/L and then decreasing at higher concentrations. The most pronounced response occurred after application in the exponential phase, when carbohydrate content at 10–30 mg/L was approximately 40–41% higher than in the control. In the stationary phase, the variations were less consistent, and at the highest concentration, carbohydrate content decreased below the control level. Two-way ANOVA revealed significant effects of concentration, application timing, and their interaction on carbohydrate content, indicating that the response varied according to both ZnO-MP concentration and the timing of application.

Lipids exhibited a distinct response profile. Following application in the lag phase, changes were moderate and non-monotonic, with significant differences among some concentrations but no consistent unidirectional trend. After application in the exponential phase, 0.5 and 5 mg/L moderately reduced lipid content, whereas 10–30 mg/L caused a sharp increase, with values reaching approximately 8.65–11.86% of biomass. A similar response was observed following application in the stationary phase, where 10–30 mg/L resulted in lipid contents of approximately 9.44–10.21%, more than twice the control level. For this parameter, two-way ANOVA also confirmed significant effects of concentration, application timing, and their interaction.

Enhanced lipid accumulation in response to ZnONPs has also been reported in microalgae. In *Chlorella vulgaris*, spherical ZnONPs at 20–50 mg/L markedly increased lipid content without significantly reducing final biomass [23], while Kaliamurthi et al. (2019) reported increased accumulation of neutral lipids and triacylglycerols in *Chlorella* sp. exposed to 50 mg/L ZnONPs [44]. A more moderate increase in lipid content was reported in *Chlorella vulgaris* at 5–50 mg/L [55]. These observations indicate that enhanced lipid accumulation may represent a recurring response to certain levels of ZnONP exposure. The magnitude and direction of this response, however, appear to depend strongly on the species and experimental system.

Taken together, the relatively limited changes in final biomass, accompanied by pronounced changes in proteins, phycobiliproteins, carbohydrates, and lipids, are consistent with biochemical remodeling and a possible redistribution of cellular resources under ZnO-MP exposure. The stimulation of phycobiliprotein and phycocyanin accumulation at low concentrations may represent either enhanced pigment accumulation or an acclimation response, whereas the increase in carbohydrates and lipids at higher concentrations may reflect altered carbon allocation associated with cellular stress. However, these mechanisms cannot be distinguished from the present compositional data alone and would require direct measurements of photosynthetic activity, oxidative stress, and related metabolic processes.

### 4.7. Concentration-Dependent Transition from Stimulation to Metabolic Stress

Integration across all endpoints reveals a concentration-dependent transition strongly modulated by ZnO-MP application timing. At the lower concentration range (approximately 0.01–1 mg/L), changes in biomass were quantitatively limited. At the same time, some biochemical components were stimulated, particularly carbohydrates following application in the lag phase and phycobiliproteins/phycocyanin following application in the exponential and stationary phases. At approximately 5 mg/L, a transition range emerged in which the response became more clearly dependent on application timing. At 10–30 mg/L, a pattern consistent with increasing biochemical stress became evident: proteins and phycobiliproteins decreased markedly, while carbohydrates or lipids accumulated under certain application conditions, and chlorophyll and carotenoids exhibited non-monotonic or stimulatory responses under specific conditions, without a generalized decline.

This profile is consistent with a biphasic response of the biological system, in which low-level exposures may stimulate certain processes, whereas higher concentrations may exceed the compensatory capacity of the cells and induce selective inhibitory effects. The literature on ZnONPs indicates that such transitions may occur at widely different concentrations among organisms, ranging from sub-mg/L levels to tens or hundreds of mg/L, reflecting the combined influence of particle size, morphology, aggregation, medium composition, and cellular physiology [5,6,7,43,49]. For this reason, the term hormesis should be used with caution; the present data demonstrate a biphasic concentration–response pattern for certain endpoints but are insufficient to establish a general hormetic response.

This interpretation is further supported by the MDA and ABTS results. At higher ZnO-MP concentrations, increased MDA accumulation was generally accompanied by decreased antioxidant activity, indicating enhanced lipid peroxidation and a reduced antioxidant capacity of the aqueous biomass extracts. In contrast, at lower concentrations, MDA remained close to or below the control level under several application conditions, consistent with a more limited oxidative impact. Oxidative stress has also been implicated in the response of photosynthetic microorganisms to ZnO nanomaterials [22,25,30,41]. Thus, the MDA and ABTS responses provide additional evidence that the transition observed at higher ZnO-MP concentrations is associated with increasing oxidative stress. However, because ROS production and specific antioxidant defense systems were not measured directly, the underlying oxidative mechanisms cannot be established.

The mechanisms underlying these responses to ZnO-MPs were not directly investigated in the present study. Previous studies have attributed the biological effects of ZnONPs to a combination of particle–cell interactions; alterations in redox status; disruption of photosynthetic and metabolic processes; and, depending on the exposure medium, the contribution of dissolved zinc. In the present system, the relatively limited magnitude and non-monotonic pattern of biomass changes, together with the high sensitivity of proteins and phycobiliproteins and the selective responses of chlorophyll and carotenoids, are consistent with differential biochemical remodeling rather than with generalized, progressive growth inhibition. Confirmation of the underlying mechanisms will, however, require direct measurements of ROS, lipid peroxidation markers, antioxidant status, intracellular zinc, and/or the dissolved Zn fraction.

The concentration- and application timing-dependent response patterns across the investigated endpoints are summarized in Figure 7.

### 4.8. Biological Significance and Biotechnological Implications

From a biological perspective, the most important result is that the relatively limited changes in biomass do not imply a limited biological effect of ZnO-MPs. Although final biomass was significantly affected by concentration, application timing, and their interaction, its variation remained considerably smaller than the changes observed in biochemical composition. Within the same experimental range, individual biochemical components exhibited responses ranging from selective stimulation to pronounced reduction, depending on concentration and application timing. Therefore, evaluating the effects of nanomaterials on *Limnospira platensis* should include multiple biochemical endpoints and consider the timing of exposure in relation to the physiological state of the culture.

The present concentration range was selected to characterize concentration-dependent biological responses under controlled cultivation conditions and should not be interpreted as representative of ZnO exposure levels in natural aquatic environments. In particular, responses observed at the higher experimental concentrations cannot be directly extrapolated to freshwater or wastewater systems, where ZnO/Zn exposure levels, speciation, and physicochemical conditions may differ substantially. Accordingly, the present findings primarily describe ZnO-MP–cyanobacterium interactions under controlled cultivation conditions rather than predict ecological effects.

This dependence also has potential biotechnological implications. Concentrations of 0.1–1 mg/L applied in the exponential or stationary phases promoted the accumulation of phycobiliproteins and phycocyanin without a substantial reduction in biomass, whereas higher concentrations applied in the exponential phase favored carbohydrate accumulation, and 10–30 mg/L applied during the exponential or stationary phases substantially increased lipid content. These results suggest that ZnO-MP concentration and application timing could potentially be used to modulate the biochemical composition of the biomass toward specific components. Such an approach would require careful definition of an appropriate response window, since the same concentrations that stimulate a particular component may simultaneously cause a substantial reduction in another.

Overall, the present study shows that the response of *Limnospira platensis* to ZnO-MPs is determined by the interaction between nanomaterial concentration and application timing. The multipod morphology may contribute to the distinct response profile observed relative to that previously reported for spherical ZnONPs, although this possibility requires direct comparative validation under identical experimental conditions. At the same time, the differential sensitivity of proteins, phycobiliproteins, carbohydrates, lipids, and photosynthetic pigments highlight a complex biochemical remodeling that can occur despite relatively limited and non-monotonic changes in final biomass. This perspective emphasizes the importance of considering biochemical composition alongside biomass production when assessing both the biological effects and potential biotechnological applications of ZnO nanostructures.

## 5. Study Limitations and Future Directions

Several limitations should be considered when interpreting the present results. First the experimental design was intended to evaluate the effect of ZnO-MP application timing during culture development at a common final harvest point rather than to isolate physiological growth phase as an independent causal factor. Because ZnO-MPs were applied at 0, 72, and 120 h and all cultures were harvested at 144 h, application timing was necessarily associated with different exposure durations. Thus, the observed differences reflect the combined influence of the physiological state at exposure initiation and exposure duration and should not be interpreted as evidence of intrinsic phase-specific sensitivity. Future experiments using equal exposure durations initiated at different physiological stages would help distinguish these effects.

Although ZnO-MPs were characterized by SEM and XRD, and Zn release was evaluated in the culture medium, several aspects of their behavior under cultivation conditions were not directly quantified, including specific surface area, zeta potential, aggregation, sedimentation, and surface transformations. Moreover, the ultrafilterable Zn fraction does not specifically represent free Zn^2+^, and the absence of a soluble Zn^2+^ control prevents a clear distinction between particle-associated and ionic effects. Further physicochemical characterization together with appropriate ionic controls would therefore improve the mechanistic interpretation of ZnO-MP–cyanobacterium interactions.

The fate of ZnO-MPs after their addition to the cultures was not investigated. Their partitioning between the culture medium and cyanobacterial biomass, association with the cells, and recovery after cultivation remain unknown. Future studies combining electron microscopy of exposed cells with quantitative Zn determination in the biomass and culture medium would help clarify particle–cell interactions and ZnO-MP fate.

The biochemical analyses focused on selected major biomass components and were not intended to provide a complete compositional mass balance. Because the individual components were quantified independently using method-specific assays and calibration standards, their combined values should not be interpreted as accounting for the entire biomass dry weight. Other cellular and inorganic constituents were not characterized. Although the multipod morphology of the synthesized ZnO material was characterized by SEM, ZnO nanostructures with different morphologies were not compared under identical experimental conditions. Accordingly, the contribution of multipod architecture to the observed biological responses cannot be separated from other material- and exposure-related factors. Direct comparative studies using ZnO materials with controlled differences in morphology would be required to establish morphology-specific effects.

## 6. Conclusions

This study demonstrates that the biological response of *Limnospira platensis* to multipod ZnO nanostructures is strongly dependent on both concentration and application timing and cannot be adequately characterized by changes in biomass alone. Across the tested concentration range, final biomass showed relatively limited and non-monotonic variation, whereas the biochemical composition of the biomass underwent substantially greater changes.

Proteins and phycobiliproteins, particularly phycocyanin, were among the most responsive components. Low ZnO-MP concentrations stimulated total phycobiliprotein and phycocyanin accumulation under specific application conditions, whereas higher concentrations caused pronounced depletion, especially following later application. Elevated ZnO-MP concentrations also promoted carbohydrate and/or lipid accumulation under specific application conditions, while chlorophyll and carotenoids showed more moderate and non-monotonic responses. These contrasting patterns indicate differential sensitivity of biomass components to ZnO-MP exposure rather than a uniform biochemical response.

Overall, the findings identify concentration and application timing during culture development as interacting determinants of the response of *Limnospira platensis* to ZnO-MPs and demonstrate the importance of evaluating multiple biochemical endpoints alongside biomass. The observed response windows may also be relevant to the controlled modulation of cyanobacterial biomass composition. Further studies separating the physiological stage from exposure duration and incorporating mechanistic endpoints are needed to clarify the processes underlying these responses.

## Figures and Tables

**Figure 1:**
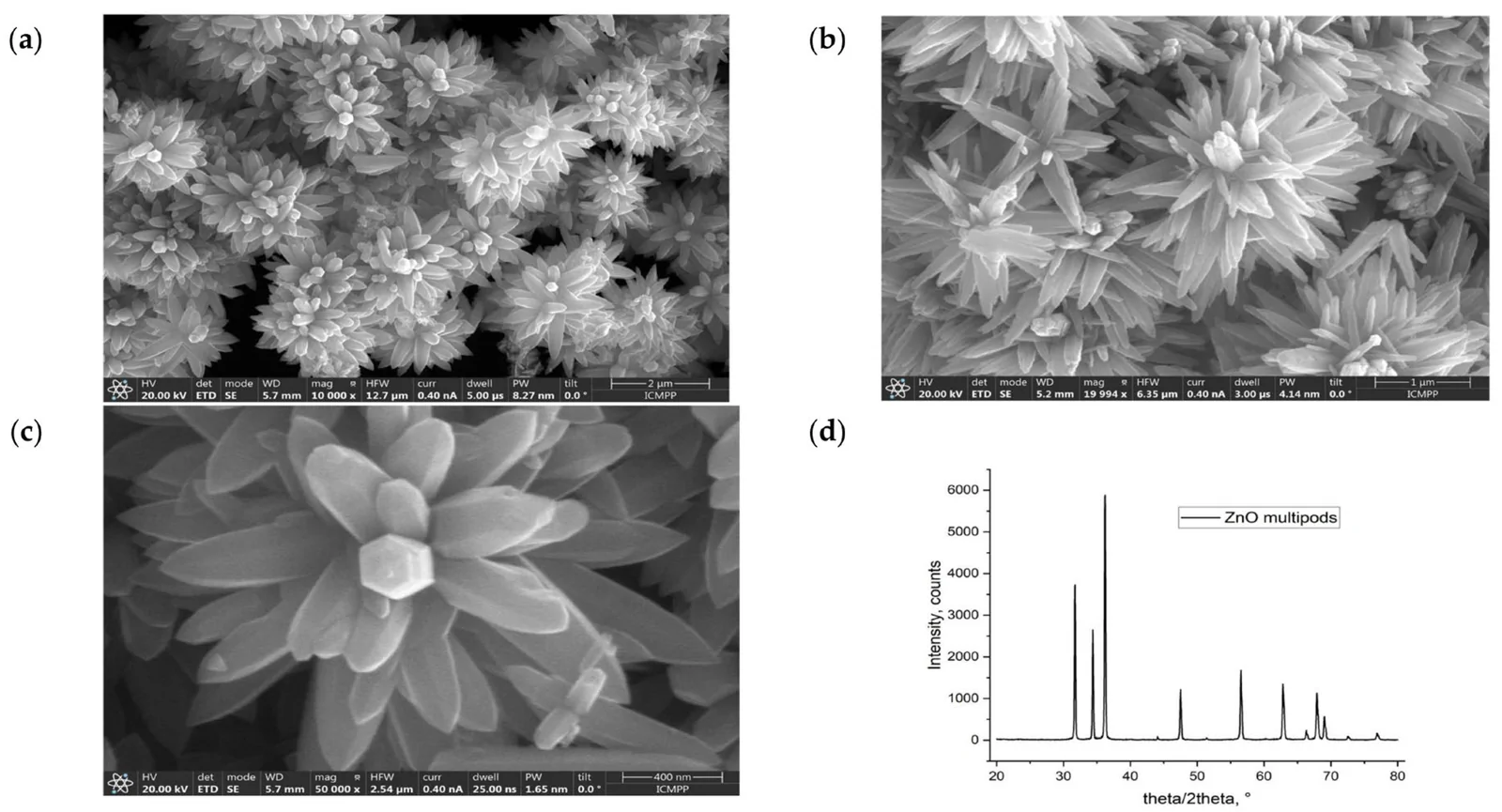
Morphological and structural characterization of the synthesized ZnO multipod nanostructures (ZnO-MPs). (**a**–**c**) SEM micrographs at increasing magnification: (**a**) overview showing the aggregation of multipod structures; (**b**) characteristic flower- or rosette-like multipod architecture formed by radially arranged elongated ZnO units; (**c**) high-magnification image showing the rod- or lance-like morphology of the individual elongated units forming the multipod structures. Scale bars: 2 μm (**a**), 1 μm (**b**), and 400 nm (**c**). (**d**) X-ray diffraction (XRD) pattern of the synthesized ZnO-MPs, showing the characteristic reflections of the hexagonal wurtzite ZnO structure (space group P6_3_mc; JCPDS card No. 36-1451).

**Figure 2:**
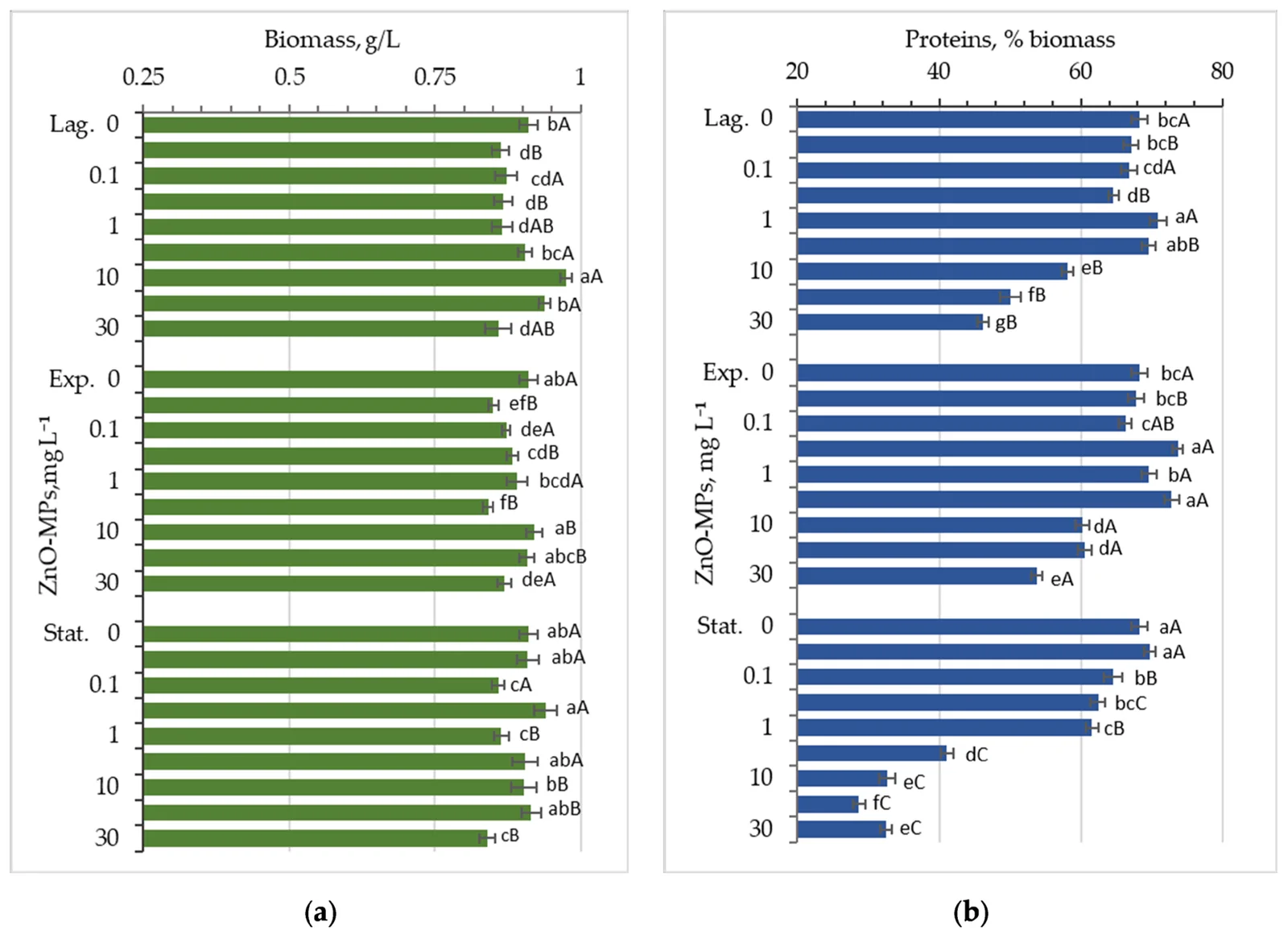
Effects of ZnO-MP concentration and application timing on final biomass (**a**) and protein content (**b**) of *Limnospira platensis*. ZnO-MPs were applied at 0 h (lag phase, Lag), 72 h (exponential phase, Exp.), or 120 h (stationary phase, Stat.) after inoculation. Data are presented as mean ± SD (*n* = 5 biological replicates). Different lowercase letters indicate significant differences among ZnO-MP concentrations within the same application timing, whereas uppercase letters indicate differences among application timings at the same concentration (Tukey’s HSD, *p* < 0.05).

**Figure 3:**
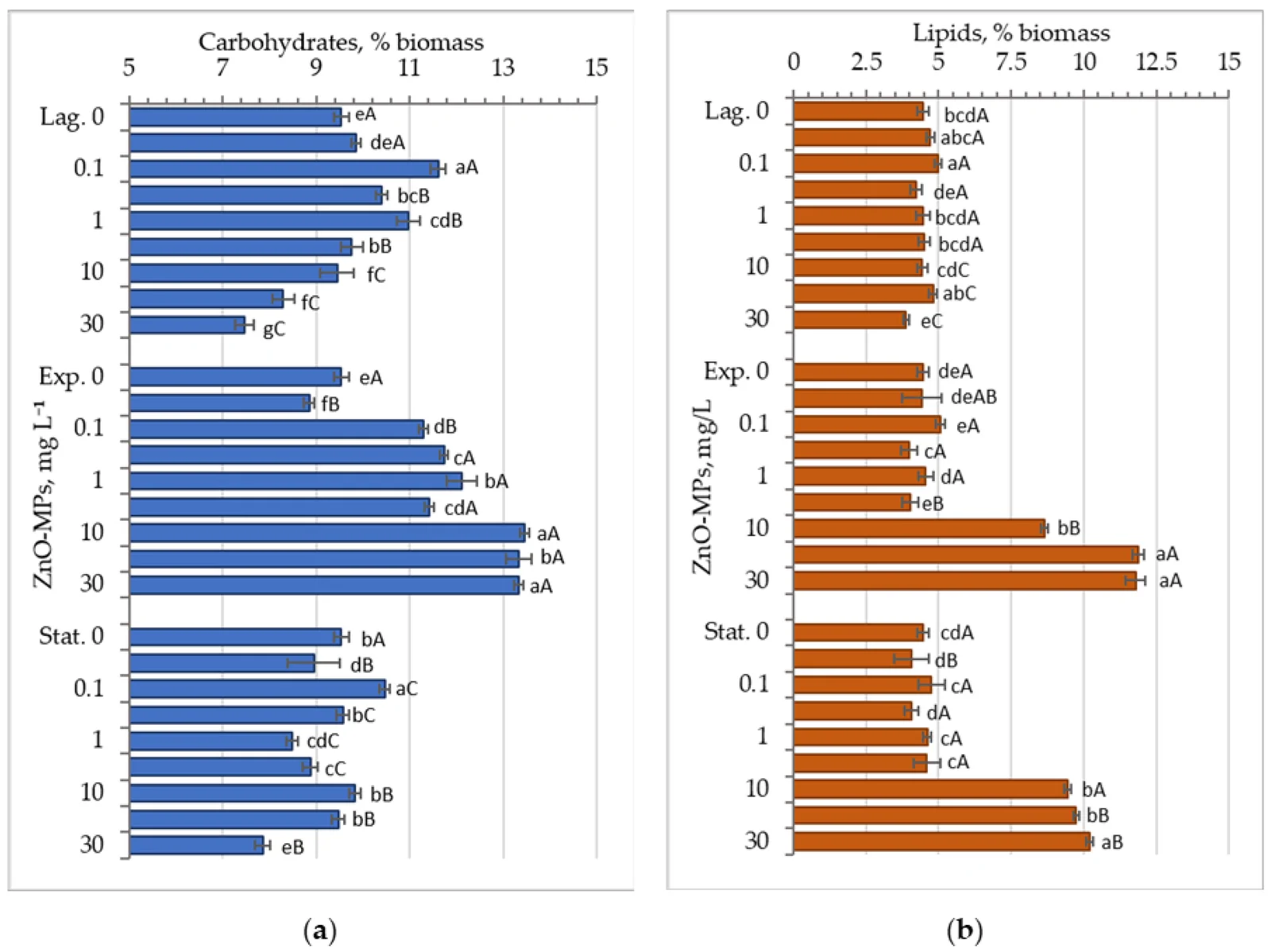
Effects of ZnO-MP concentration and application timing on carbohydrate (**a**) and lipid (**b**) contents of *Limnospira platensis*. ZnO-MPs were applied at 0 h (lag phase, Lag), 72 h (exponential phase, Exp.), or 120 h (stationary phase, Stat.) after inoculation. Data are presented as mean ± SD (*n* = 5 biological replicates). Different lowercase letters indicate significant differences among ZnO-MP concentrations within the same application timing, whereas uppercase letters indicate differences among application timings at the same concentration (Tukey’s HSD, *p* < 0.05).

**Figure 4:**
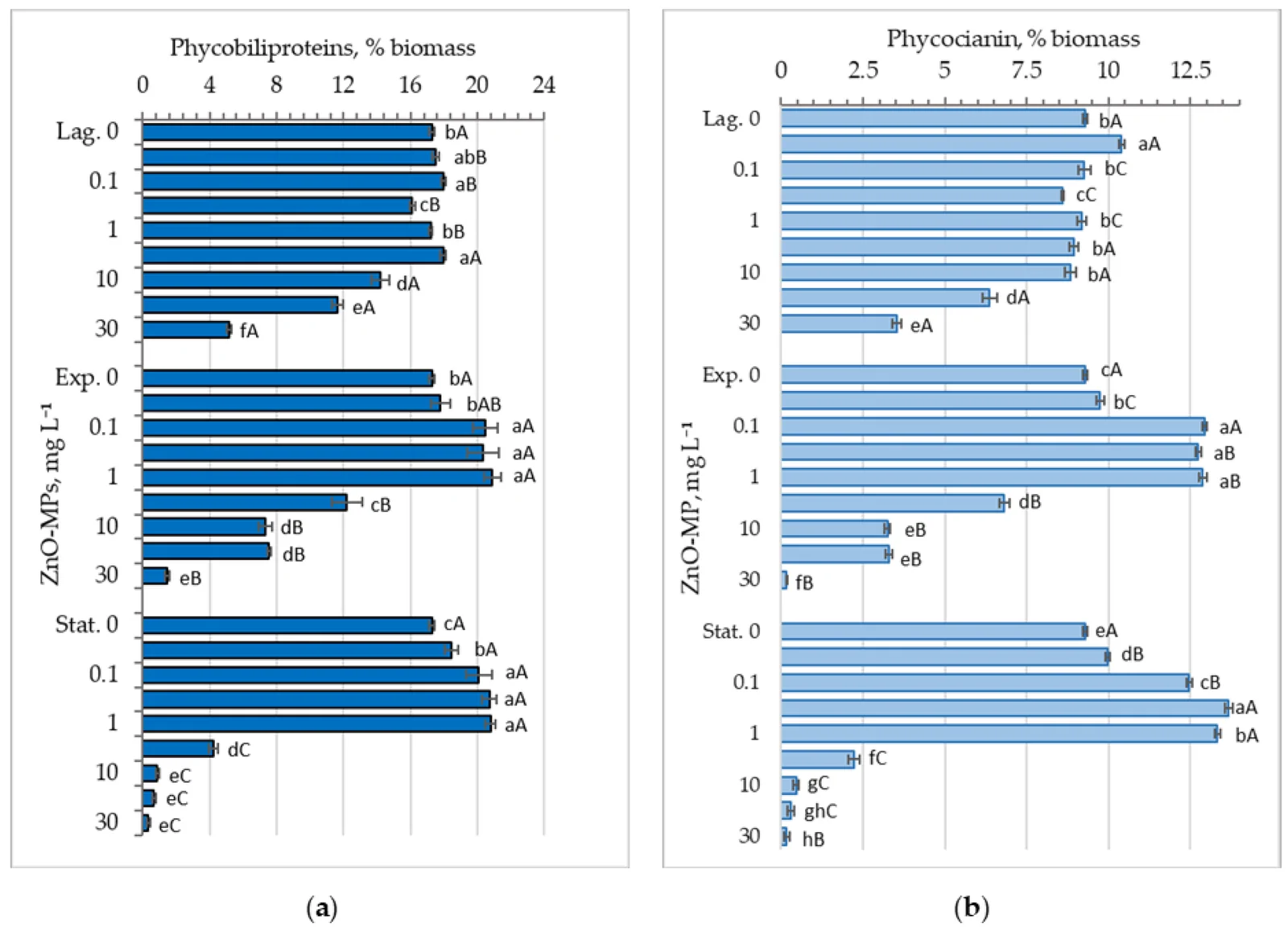
Effects of ZnO-MP concentration and application timing on total phycobiliprotein (**a**) and phycocyanin (**b**) contents of *Limnospira platensis*. ZnO-MPs were applied at 0 h (lag phase, Lag), 72 h (exponential phase, Exp.), or 120 h (stationary phase, Stat.) after inoculation. Data are presented as mean ± SD (*n* = 5 biological replicates). Different lowercase letters indicate significant differences among ZnO-MP concentrations within the same application timing, whereas uppercase letters indicate differences among application timings at the same concentration (Tukey’s HSD, *p* < 0.05).

**Figure 5:**
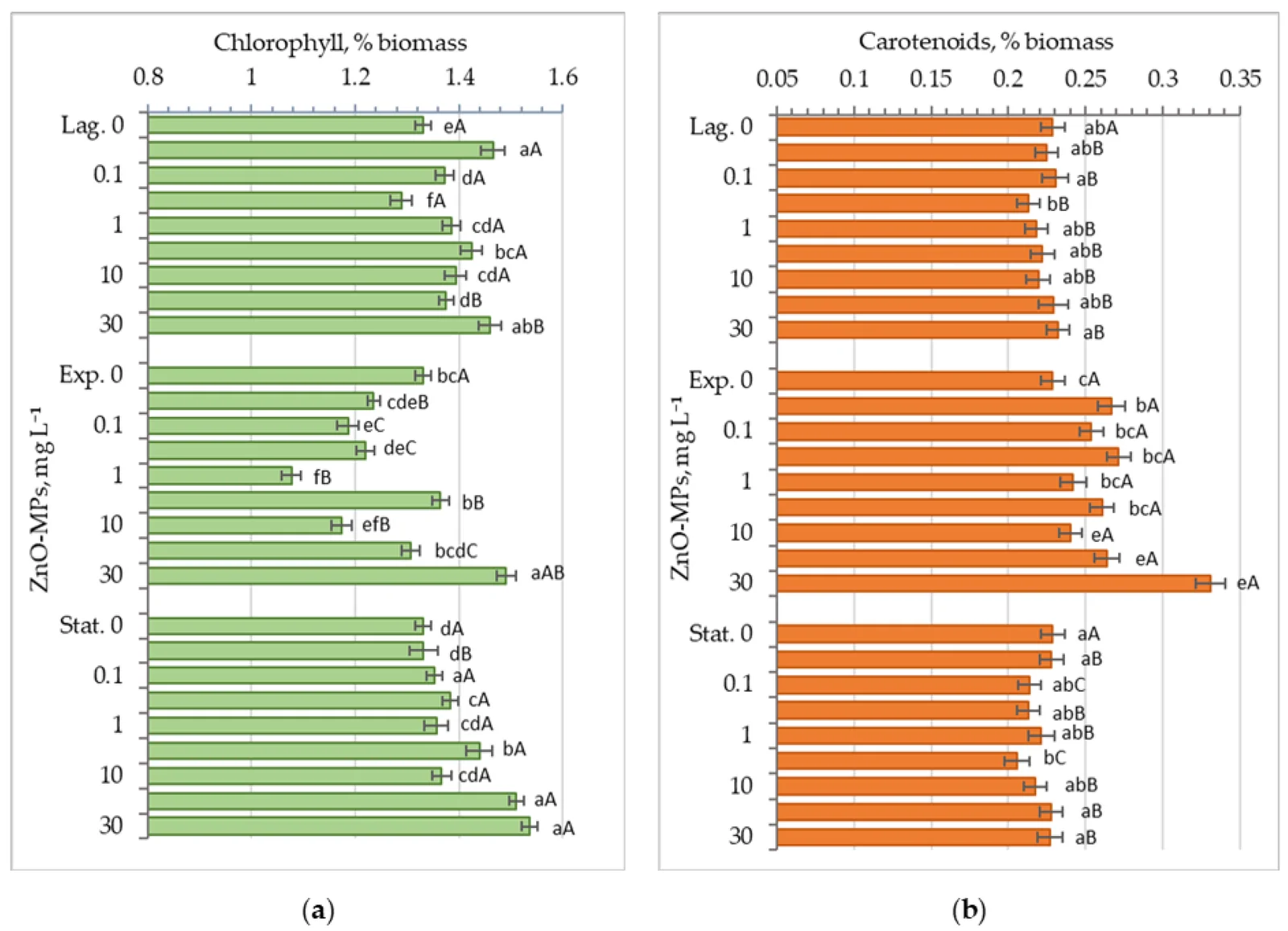
Effects of ZnO-MP concentration and application timing on chlorophyll (**a**) and carotenoid (**b**) contents of *Limnospira platensis*. ZnO-MPs were applied at 0 h (lag phase, Lag), 72 h (exponential phase, Exp.), or 120 h (stationary phase, Stat.) after inoculation. Data are presented as mean ± SD (*n* = 5 biological replicates). Different lowercase letters indicate significant differences among ZnO-MP concentrations within the same application timing, whereas uppercase letters indicate differences among application timings at the same concentration (Tukey’s HSD, *p* < 0.05).

**Figure 6:**
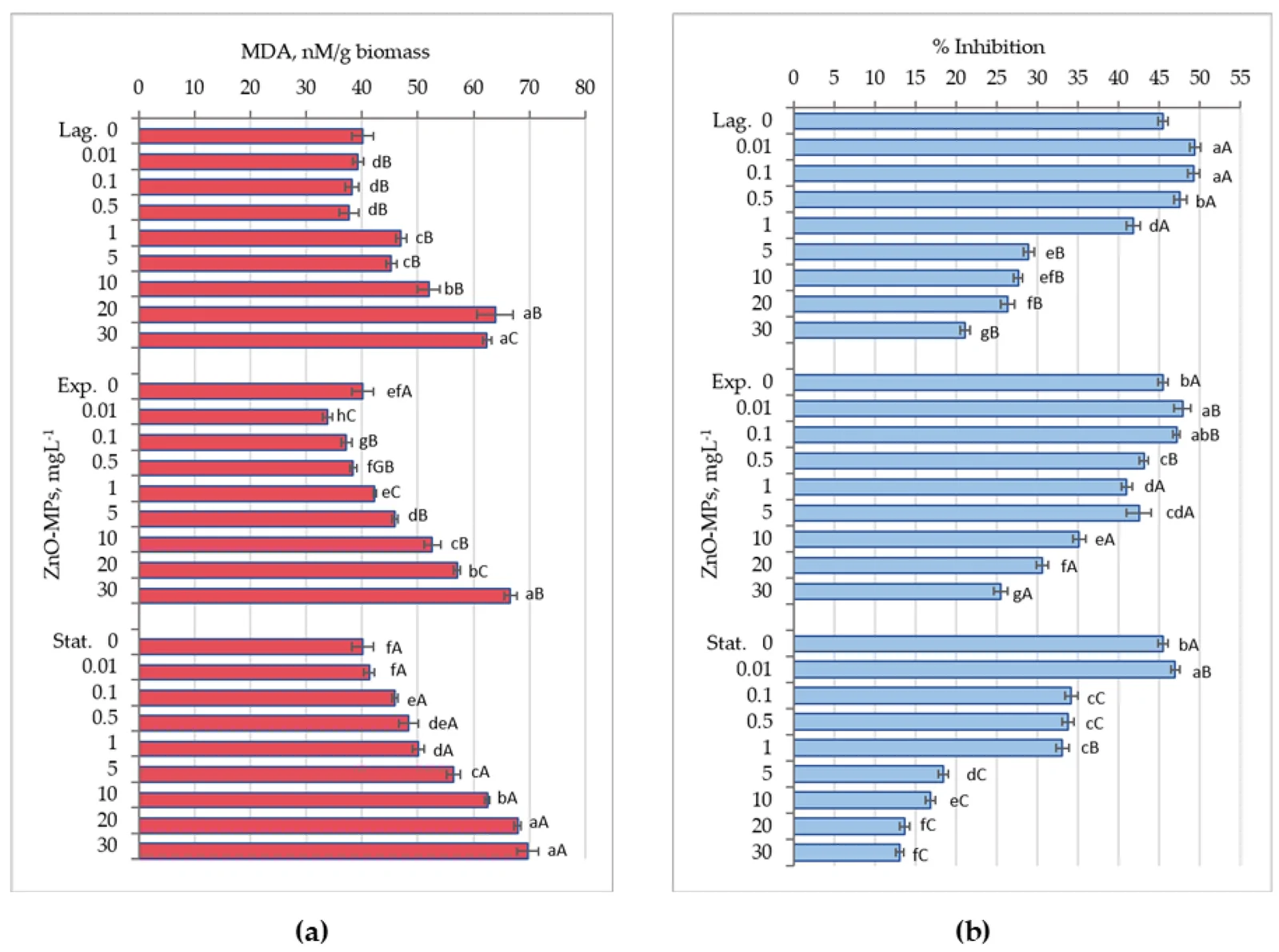
Effects of ZnO-MP concentration and application timing on malondialdehyde (MDA) content (**a**) and antioxidant activity determined by the ABTS assay (**b**) of *Limnospira platensis*. ZnO-MPs were applied at 0 h (lag phase, Lag), 72 h (exponential phase, Exp.), or 120 h (stationary phase, Stat) after inoculation. Data are presented as mean ± SD (*n* = 5 biological replicates). Different lowercase letters indicate significant differences among ZnO-MP concentrations within the same application timing, whereas uppercase letters indicate differences among application timings at the same concentration (Tukey’s HSD, *p* < 0.05).

**Figure 7:**
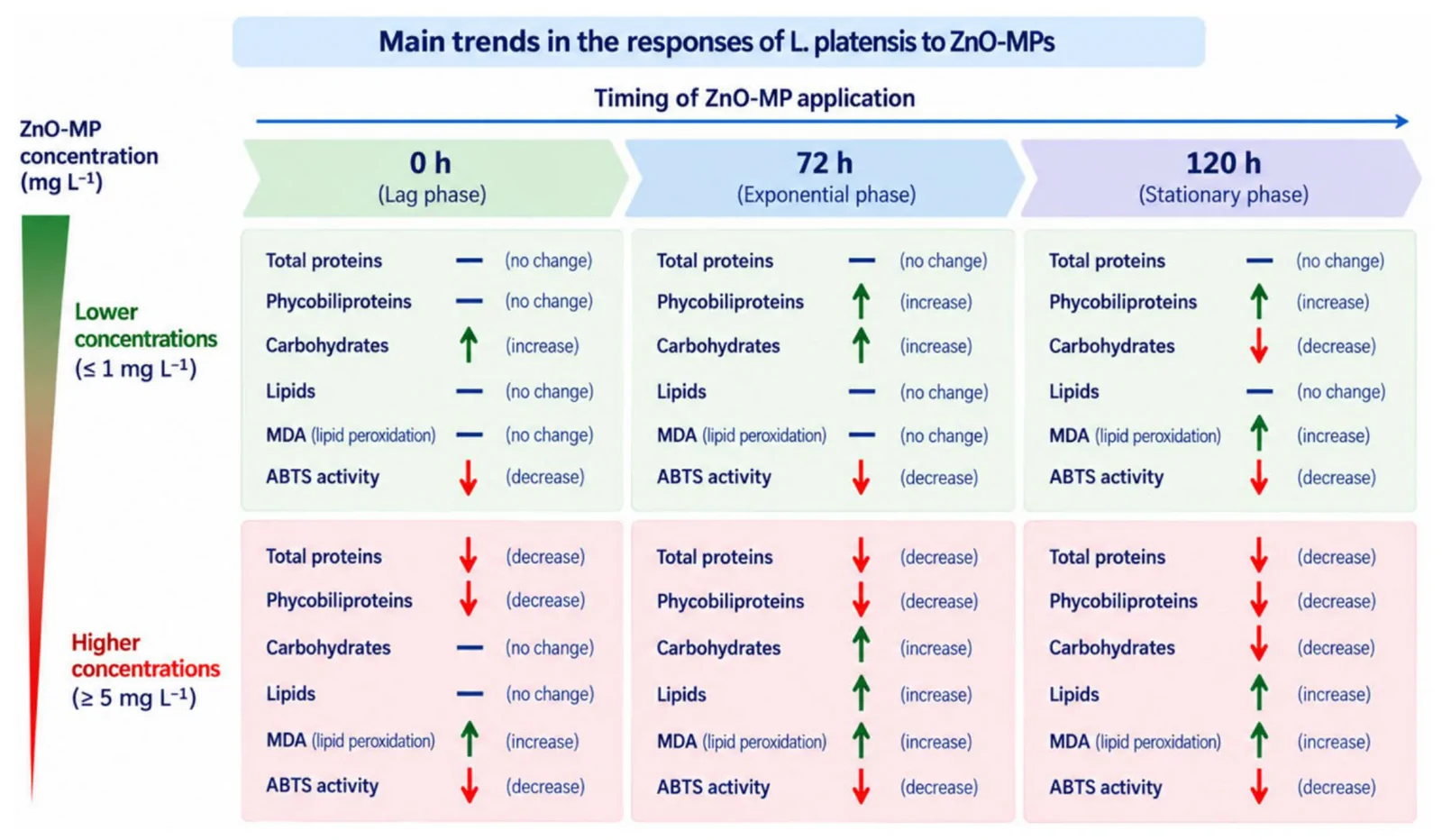
Concentration- and application timing-dependent responses of *Limnospira platensis* to ZnO-MPs. The scheme summarizes the experimentally observed response patterns across ZnO-MP concentrations and application timings (0, 72, and 120 h).

**Table 1:** Two-way ANOVA for the effects of ZnO-MP concentration, application timing, and their interaction on the analyzed endpoints *.

Endpoint	Application Timing, F(df)	Concentration, F(df)	Application Timing × Concentration, F(df)
Biomass	9.95 (2)	46.06 (7)	17.05 (14)
Proteins	3013.36 (2)	1579.05 (7)	288.16 (14)
Carbohydrates	1947.57 (2)	148.01 (7)	184.05 (14)
Lipids	1376.47 (2)	1410.33 (7)	419.38 (14)
Total phycobiliproteins	824.08 (2)	3606.23 (7)	391.96 (14)
Phycocyanin	737.15 (2)	8625.28 (7)	1346.69 (14)
Chlorophyll	299.13 (2)	68.47 (7)	24.23 (14)
Carotenoids	167.09 (2)	13.66 (7)	7.38 (14)
MDA	499.81 (2)	1006.00 (7)	16.95 (14)
ABTS antioxidant activity	3158.15 (2)	2735.13 (7)	145.20 (14)

* All reported effects were significant at *p* < 0.001.

## Data Availability

The original contributions presented in this study are included in the article. Further inquiries can be directed to the corresponding author.

## Supplementary Materials

The following supporting information can be downloaded at https://www.mdpi.com/article/10.3390/life16101604/s1. Figure S1: Growth kinetics of untreated *Limnospira platensis* CNMN-CB-02 under the cultivation conditions used in the study; Table S1: Quantitative characterization of the growth phases of untreated *Limnospira platensis* CNMN-CB-02 under the cultivation conditions used in this study; Table S2: Release of ultrafilterable Zn from ZnO-MPs in cell-free culture medium under the cultivation conditions used for *Limnospira platensis*.
