# Supplementary material for: Concentration and Application Timing Modulate Growth and Biochemical Composition of Limnospira platensis Exposed to Multipod ZnO Nanostructures
Source: Life (Basel). 2026 Sep 25;16(10):1604. doi: 10.3390/life16101604 (PMC13705868; doi:10.3390/life16101604)
Supplement: Supplementary file 1 [file life-16-01604-s001.zip › Figure S1. Growth kinetics of untreated Limnospira platensis CNMN-CB-02 under the cultivation conditions used in the study.pdf]

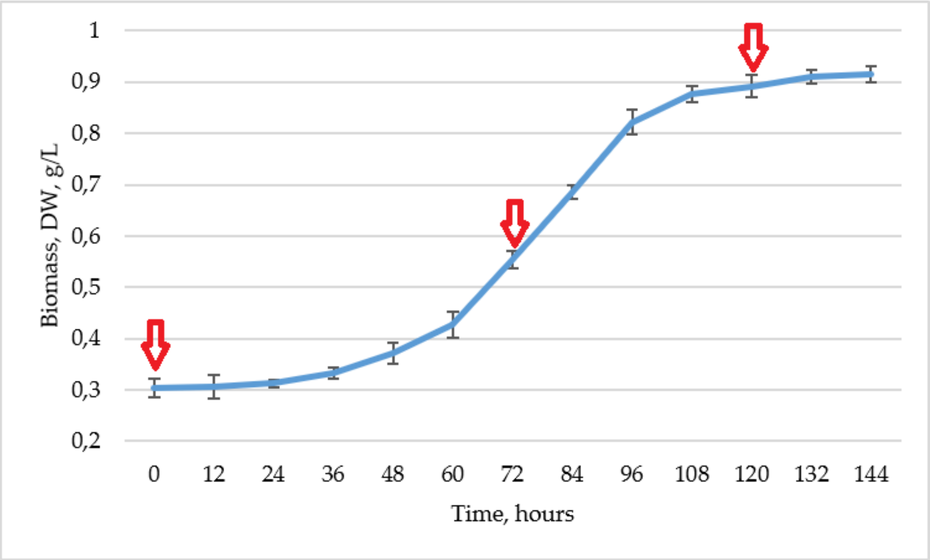

Figure S1. Growth kinetics of untreated *Limnospira platensis* CNMN-CB-02 under the cultivation conditions used in the study. Data are presented as mean ± SD from five independent biological replicates (n = 5). The experimentally identified growth phases were lag phase, 0–24 h; transition phase, 24–48 h; exponential phase, 48–96 h; deceleration phase, 96–108 h; and stationary phase, ≥108 h. ZnO-MP application time points (0, 72, and 120 h) are indicated on the growth curve.
