# Supplementary material for: Concentration and Application Timing Modulate Growth and Biochemical Composition of Limnospira platensis Exposed to Multipod ZnO Nanostructures
Source: Life (Basel). 2026 Sep 25;16(10):1604. doi: 10.3390/life16101604 (PMC13705868; doi:10.3390/life16101604)
Supplement: Supplementary file 1 [file life-16-01604-s001.zip › Table S1. Quantitative characterization of the growth phases of untreated Limnospira platensis CNMN-CB-02 under the cultivation conditions used in the study..pdf]

Table S1. Quantitative characterization of the growth phases of untreated *Limnospira platensis* CNMN-CB-02 under the cultivation conditions used in the study.

Parameter	Result
Lag phase (h)	0–24
Transition (h)	24–48
Exponential phase (h)	48–96
Deceleration (h)	96–108
Stationary phase (h)	≥108
Specific growth rate during exponential phase, μ_{exp} (regression, 48–96 h)	0,0172
R^2 of ln(biomass) vs. time regression (48–96 h)	0,9933
Doubling time during exponential phase (h)	40,28
Maximum 12-h specific growth rate, μ_{max} (h^{-1})	0,0217
Interval corresponding to μ_{max} (h)	60–72
