# Supplementary material for: Concentration and Application Timing Modulate Growth and Biochemical Composition of Limnospira platensis Exposed to Multipod ZnO Nanostructures
Source: Life (Basel). 2026 Sep 25;16(10):1604. doi: 10.3390/life16101604 (PMC13705868; doi:10.3390/life16101604)
Supplement: Supplementary file 1 [file life-16-01604-s001.zip › Table S2. Release of ultrafilterable Zn from ZnO-MPs in cell-free culture medium under the cultivation conditions used for Limnospira platensis.pdf]

Table S2. Release of ultrafilterable Zn from ZnO-MPs in cell-free culture medium under the cultivation conditions used for *Limnospira platensis*.

ZnO-MP concentration (mg/L)	Initial Zn equivalent (mg/L)	Ultrafilterable Zn concentration					Ultrafilterable Zn (% of initial Zn)
		1 h (mg/L)	6 h (mg/L)	24 h (mg/L)	72 h (mg/L)	120 h (mg/L)	
**5**	4.017	0.366 ± 0.005	0.366 ± 0.005	0.366 ± 0.005	0.366 ± 0.005	0.366 ± 0.005	9.11 ± 0.14
**10**	8.034	0.468 ± 0.016	0.468 ± 0.016	0.468 ± 0.016	0.468 ± 0.016	0.468 ± 0.016	5.83 ± 0.20
**20**	16.068	0.514 ± 0.005	0.514 ± 0.005	0.514 ± 0.005	0.514 ± 0.005	0.514 ± 0.005	3.20 ± 0.03
**30**	24.102	0.556 ± 0.005	0.556 ± 0.005	0.556 ± 0.005	0.556 ± 0.005	0.556 ± 0.005	2.31 ± 0.02

Note: Values are mean ± SD (n = 5 independent samples at each concentration and time point). The initial Zn equivalent was calculated from the Zn mass fraction in ZnO (65.38/81.38 = 0.8034). The percentage of ultrafilterable Zn was calculated relative to the total elemental Zn initially present in the added ZnO-MPs. Identical mean values across 1–120 h reflect the absence of a measurable time-dependent increase under the tested conditions.
